# Complete Genome Sequence of a New Megavirus Family Member Isolated from an Inland Water Lake for the First Time in India

**DOI:** 10.1128/genomeA.00402-16

**Published:** 2016-06-16

**Authors:** Anirvan Chatterjee, Farhan Ali, Disha Bange, Kiran Kondabagil

**Affiliations:** Department of Biosciences and Bioengineering, Indian Institute of Technology Bombay, Powai, Mumbai, India

## Abstract

We report here the isolation and complete genome sequencing of a large double-stranded DNA virus, *Powai Lake megavirus,* for the first time from India. The isolation of a large DNA virus with genome size >1 Mb from India further attests to the prevalence of *Giant* viruses in different environmental niches.

## GENOME ANNOUNCEMENT

Nucleocytoplasmic large DNA viruses (NCLDVs), or *Giant* viruses, have genome sizes reaching up to 2.5 Mb (1) and include four families. With their large unique genomes and widespread presence in the aquatic environments (2–6), the NCLDVs are thought to be one of the major vehicles of evolution and are now being explored to revisit evolutionary paradigms, such as origin of eukaryotes (7), evolution of DNA replication system (8), genome packaging systems (9, 10), etc. Sequencing of more NCLDV genomes is necessary to understand their ecological and evolutionary significance.

In this study, we isolated and sequenced the genome of a large double-stranded DNA virus infecting a free-living amoeba, *Acanthamoeba castellanii*. Water samples were collected from Powai Lake, an artificial inland lake in Mumbai, India, and processed for isolation of large viruses against the amoeba host, as per published protocols (11). Transmission electron microscopy revealed icosahedral particles of about 425 nm in diameter comparable to that of some NCLDVs reported previously. Isolated virus was propagated in *Acanthamoeba castellanii*, purified on a sucrose gradient, and the genome was extracted as described earlier (11). Whole-genome shotgun sequencing was performed using Illumina MiSeq 2 × 150-bp paired-end chemistry that yielded 3,481,650 reads. Kraken metagenomics (Illumina BaseSpace webtool), performed with trimmed and quality control (QC)-filtered reads, taxonomically classified 15% of the reads as *Megaviridae*; hence, the isolate was named *Powai Lake megavirus* (PLMV). The G+C content of PLMV (25%) is comparable to that of other *Giant* viruses.

*De novo* assembly was performed using A5-miseq (12) and evaluated using QUAST (13). The PLMV assembly exhibited a median coverage of 793×, with an *N*_50_ value of 750,973 bp. All contigs were aligned to the BLAST NR database using MegaBLAST (14, 15), and a consensus FASTA sequence was generated by reordering the 16 contigs using MAUVE (16). The PLMV genome size was found to be 1,208,707 bp, with 996 open reading frames (ORFs), as predicted by GeneMarkS (17). The ORFs were individually annotated using BLASTP (15), and the results were retrieved using custom Python scripts. The annotated genomes were uploaded to NCBI using BankIt Web-based submission tool. Using tRNAscan-SE (18), PLMV was found to encode 5 tRNAs, and the CRISPRFinder Web tool (19–21) detected 3 confirmed clustered regularly interspaced short palindromic repeats (CRISPRs) and 6 CRISPR-like sequences in the PLMV genome. Further, a TransposonPSI (http://transposonpsi.sourceforge.net/) search yielded at least 4 hits in the PLMV genome. The isolation and whole-genome sequencing of a first NCLDV from India presents an opportunity to study their significance in India’s rich ecological diversity.

### Nucleotide sequence accession number.

The complete genome of PLMV has been deposited in the GenBank under the accession no. KU877344.
